# Antiseptic Surgery

**Published:** 1883-03

**Authors:** W. S. Tremaine

**Affiliations:** Major and Surgeon, U. S. A.


### BUFFALO

### Medical and Surgical Journal.

Vol. XXII. MARCH, 1883. No. 8.

### Original Communications.

### Antiseptic Surgery.

By W. S. Tremaine, M. D.,

Major and Surgeon, U. S. A.

Antiseptic surgery has now been on trial for about ten years, and with such success that one might infer that it had been generally adopted and formed a well-recognized surgical proceeding. While this may be true of our larger cities, with their well-appointed hospitals, abundant opportunity for observation has taught me that antiseptic surgery, properly so-called, is not intelligently used by the average practitioner, and, indeed, neglected in some hospitals, to the manifest increase of mortality in surgical injuries. True, antiseptic agents are applied to wounds, but often to their detriment, and without any clear idea of the purpose for which they should be used, or with a vague impression that they in some way promote healing. Modern pathology, confirmed by clinical observation, has taught us that inflammation is not necessary to the repair of wounds, but, in fact, retards and interferes with it; therefore, the application of a strong antiseptic agent, while it possibly prevents sepsis, may irritate and inflame, and thus delay the healing process. The inconvenience from this would be of slight moment as compared with the grave danger to life caused by the decomposition of the exudates in wounds. It is this recognition of the direful results from sepsis that has impelled surgeons to seek some process by which they may be prevented, even at the risk of retardation of the healing process, or of a small amount of irritation. It is against the results of septic infection that antiseptic surgery, as such, is directed, and not primarily, as has been supposed by some, to promote the healing of wounds.

That form of surgical dressing which will, in addition to perfect asepsis, prevent the other accidents to which wounds are liable, for instance, inflammation, tension, etc., must, by all, be admitted to be desirable, and to this end is directed the earnest effort of practical surgeons.

The writer has, for the past ten years, given especial attention to this subject, and as surgeon, for six years, to one of the great trans-continental railroads, and, later, while professor of surgery in Kansas City (a large railroad centre), has had extended personal experience in the treatment of wounds, both operative and accidental, which he believes is sufficient warrant for presenting his views in this way.

The great dangers consequent upon putrefactive changes in the discharge from wounds have long been recognized, and various methods have been, from time to time, adopted, with a view to lessen or prevent them. That some deleterious influence from the air was the most potent factor in the production of these putrefactive changes was apparent. The difference in the behavior of a subcutaneous lesion and an open wound early attracted attention. A marked illustration of this is to be seen in the contrast between a simple and compound fracture, and as it became evident that some deleterious agent derived from the atmosphere brought about decomposition in the exudates from wounds, the method of treatment by occlusion was practiced, and of this one of the best was that of Guerin by the use of cotton-wool. This proved to be of great advantage in a number of cases of lacerated and gun-shot wounds. After a thorough washing with dilute alcohol, the cotton-wool was liberally applied and retained by a bandage, and in many cases allowed to remain without change until the wound healed. Notably, an excellent result was obtained by this method in a case where I amputated both arms and legs for frostbite and reported in the *New York Medical Journal* for January, 1882. In 1876 my attention was brought to the subject of Listerism. From that time to the present I have had a large personal experience in the use of this method of dressing, and have also had extended opportunities of seeing its practice and results in the hospitals of some of our large cities, and I must give it my decided approval; and this leads me to attempt to describe what is meant by Listerism.

Again and again I have heard surgeons say: "I have tried Listerism and do not approve of it." A closer examination of their method of practicing what they *called* Listerism, convinced me that they had but a faint idea of the process advocated and taught by Mr. Lister. For instance, I have seen ordinary adhesive plaster applied in strips, and then the dry, carbolized gauze put on, and *over* this the "protective"! Now, while this *may* be a form of antiseptic dressing, it is certainly *not* Listerism.

Again, I have seen an eminent surgeon (who informed me that he practiced Listerism but did not get the results claimed for it) deliberately lift up the dressings from a patient in a crowded ward of a hospital and replace them without the protection of the spray!

He who would successfully practice Listerism must believe in the "germ theory," for the practice is the logical outcome of this theory, and is not the application of any special therapeutic agent to a wound. It is, in short, a method of surrounding a wound with an antiseptic atmosphere while providing for the escape of the discharge. The essentials are these:

1st. Operating in an antiseptic atmosphere (produced by the spray), or if the wound be accidental (as distinguished from operative), making it aseptic by the *thorough* use of a strong solution of carbolic acid (1 to 20), or of chloride of zinc (10 per cent).

2d. The prevention of any contact of septic materials, accomplished by using only *antiseptic* hands, instruments, ligatures, drainage tubes, sutures, etc.

3d. The prevention of the future access of germs by covering the wound with an antiseptic absorbent material (the gauze).

4th. The protection of the latter from germs when saturated with discharge by the use of an impervious covering (the Mackintosh or gutta percha).

5th. Physiological rest by infrequent removal of the dressings.

6th. The prevention of irritation from the dressing itself by the use of the "protective" between the gauze and the wound.

These, then, are the conditions of a complete surgical dressing, to place the wound in the best possible condition for healing, and to prevent the serious consequences that ensue from decomposition of the discharges. If Listerism does this, and without failure, then it is the best form of surgical dressing, and should be invariably used. But what are the objections to its use?

1st. It involves considerable paraphernalia.

2d. A thorough acquaintance with numerous minute details, a careful attention to which is essential to its success.

Objections which should have no weight if it can be conclusively shown that the process in its entirety is absolutely necessary to the complete protection of wounds. This is the essential question. If the germ theory is correct, then the principles of Listerism are necessary.

The question of the most effective and practical method and materials to carry out these principles is to be determined by experience, and, let me here say, chiefly, if not altogether, by *clinical* experience.

As usual when any new method has been generally successful, attempts are vigorously made to discredit the advantages, or to explain the benefits derived upon some other grounds. When this takes the form of a healthy and honest criticism, it is certainly most desirable. Accordingly, from time to time efforts have been made to disparage Listerism. The inconvenience of, and occasional danger of systemic poisoning from the carbolized spray has made it desirable that it should be omitted if it can be done with safety. There are various opinions in regard to this. My own is that where an operation is performed in what is believed to be a fairly aseptic atmosphere, that the spray may be dispensed with, and irrigation, after the operation is completed and before the dressings are applied, substituted; in short, as in all other matters, the necessity for the spray to be determined in each case by the judgment of the surgeon. In *redressing*, and particularly when done in a surgical ward, the spray should be invariably used.

For antiseptics to impregnate gauze, in lieu of carbolic acid, thymol and eucalyptus have been used; they have not proved satisfactory in my hands.

In 1878 my accomplished friend, Surgeon B. E. Fryer, U. S. A., Professor of Opthalmology in Kansas City, brought to my notice the value of iodoform as an antiseptic dressing, and I think he is entitled to credit for priority of its use in this way. My personal experience with it is limited, but I believe in properly-selected cases it is valuable, non-irritant, and locally an anæsthetic, in this way vastly superior to carbolic acid. From its unirritating qualities, it permits of the omission of the "protective," and being less volatile than carbolic acid, the "Mackintosh" can be dispensed with, so far simplifying the dressing.

But the objections urged against carbolic acid, viz., the production of poisoning, apply, perhaps, in a greater degree to iodoform, and the report comes from Europe that even its use in small quantities is attended with danger from toxæmia, with the addition of erysipelas.

All this demonstrates that a perfect antiseptic has not yet been discovered, nor, for that matter, has a perfect anæsthetic. No one, however, would refrain from using ether because an occasional death occurred. One of the disadvantages of the Lister system that I early noticed was the rubber drainage tube. This, after a few days, acts as a seton, and should be removed, requiring a renewal of the dressings, always a drawback unless absolutely required to prevent wound accidents. I soon, therefore, omitted it in superficial wounds, or substituted carbolized cat-gut. The introduction of the decalcified bone tubes of Neuber is, in my judgment, a marked improvement. In a case where I amputated at the middle third of the leg, two of these decalcified chicken-bone tubes were used, the ordinary gauze layers applied, and over all a pad or cushion of the carbolized "jute," suggested by Dr. Weir, of New York, this covered by gutta percha tissue. But one dressing in all was applied, and on the twelfth day the man was walking about on crutches, with his stump completely healed. In another case, removal of a large lipoma from the region of the scapula, one of these bone tubes was used and but one dressing from first to last. Where the rubber drainage tubes are used it is a frequent practice to syringe them out with strong carbolized water, for the purpose of cleaning them. This is wrong. Where there is no evidence of sepsis in the wound the tubes should not be disturbed, beyond freeing their lumen from clots, etc., with an ordinary aseptic probe. Constant efforts are being made to improve and simplify the details. Among the latest ones may be mentioned the improved cat-gut ligature described in the *Lancet* for March, 1882, using chromic acid and sulphurous acid, requiring only a few hours for its preparation. This cat-gut has the advantage of being stronger, and it can be carried about dry, only needing to be soaked in carbolized water for a short time before using. One objection urged against the carbolized gauze is that it is not easily obtained by the country practitioner. Messrs. Seabury & Johnson, of New York, now manufacture a superior article, enveloped in waxed paper and put up in air-tight tin cans of convenient size, which can be kept on hand till needed. Or, if the surgeon prefers to make his own gauze, this can be readily done by keeping on hand a quantity of the fabric known as "cheese cloth," rendered hydroscopic by boiling in a weak solution of caustic soda. This can be rendered antiseptic by soaking it in the following mixture, which is the formula of Brun: Resin, 400 grammes; carbolic acid, 100 grammes; castor oil, 80 grammes; alcohol, 2 litres. Or, the the jute recommended by Dr. Weir can be used.

In the smaller class of wounds my usual practice is to wash them thoroughly with a solution of carbolic acid, three per cent., close them accurately with antiseptic cat-gut sutures, sprinkle a little iodoform on the wound, then apply a pad of salycilic cotton, over this a piece of gutta percha tissue, the whole secured by a few turns of bandage. A wound dressed in this way rarely requires a second dressing before it is healed. The freedom from pain and annoyance of frequent dressing delights the patient and saves the busy surgeon much unnecessary trouble.

During the past summer my attention was attracted to boroglyceride, brought to notice by Prof. Barff, of London, in March last. I immediately prepared some, and through the kindness of my friend, Dr. Phelps, the attending surgeon at the Buffalo General Hospital, was enabled to test it upon some cases in the hospital. The results were excellent, the wounds healing rapidly without pain or the slightest irritation. Dr. Mann, the accomplished Professor of Obstetrics at the Buffalo University, has also used this agent with excellent results in ovariotomy. Mr. Barwell, of London, publishes a report of twelve cases, embracing major amputations and compound fractures, giving results that promise much for this new claimant for antiseptic honors.

In conclusion, I am decidedly of the opinion that a failure to use antiseptics in surgical practice savors strongly of criminal negligence. In hospitals they are indispensable. In properly-selected cases that form of antiseptic surgery known as Listerism most surely protects against the dangers from sepsis. That where it fails, the failure is not due to the method itself, but either to the imperfections in its application or to the inapplicability of the system of Lister to the case, which occasionally happens. Take, for instance, an extensive lacerated wound into which innumerable particles of dirt are imbedded beyond the reach of absolute and complete removal, and where portions of tissue are so injured that they are incapable of becoming organized. In such a case Listerism will frequently fail, but even then no harm can result from making the attempt. In two cases of compound gun-shot fracture ot the femur, which I treated by Listerism, both recovered in less than six weeks with a very slight amount of suppuration and absolutely no constitutional irritation. In one of the tibia, compound and comminuted, while there was necrosis and suppuration, there was no sepsis and no rise of temperature. Did time and space permit, I could give case after case in illustration of the value of antiseptic surgery occurring within my personal observation, but I turn to the weight of greater authority. In a list of cases published in the sixth congress of German surgeons there are seventy-five compound fractures; all recovered. There were forty-two amputations of the thigh, with but one death; fifty osteotomies, with but one death, and that in a "bleeder." Schede gives thirty-seven compound fractures with one death. Dr. Weir gives forty major amputations, without a single death; also, a consecutive list of seventy compound fractures without a death; and in 150 major compound fractures, only three deaths. Contrast this with seventeen consecutive cases of compound fracture, all fatal, in a hospital where Listerism is not used.

The idea is advanced by some that while antiseptics may be necessary in hospitals, they are not needed in private practice. For the sake of argument, admit this, and as compound fractures can be better treated, as a rule, in all other respects, in a hospital, is it not imperative that the increased dangers from sepsis should be rigidly guarded against by the invariable use of that method which experience has shown to be best for the purpose. A properly-managed hospital, with a thoroughly competent staff, is a great boon to the poorer classes; but it *can* be made a *pest* to be avoided. This, however, is leading me into a a wide subject, hospital organization and management, about which I may have something to say on a future occasion.

